# Clinical outcomes of transcatheter edge‐to‐edge repair in patients with functional mitral regurgitation and pulmonary hypertension

**DOI:** 10.1111/eci.70130

**Published:** 2025-10-03

**Authors:** Alessandro Mandurino‐Mirizzi, Luca Raone, Andrea Raffaele Munafò, Fabrizio Gazzoli, Marco Mussardo, Claudio Montalto, Francesco Germinal, Marco Ferlini, Italo Porto, Giuseppe Colonna, Jacopo Oreglia, Gabriele Crimi

**Affiliations:** ^1^ Division of Cardiology “Vito Fazzi” Hospital Lecce Italy; ^2^ Department of Experimental Medicine (DiMeS) University of Salento Lecce Italy; ^3^ Division of Cardiology 1 Fondazione IRCCS Policlinico San Matteo Pavia Italy; ^4^ Interventional Cardiology De Gasperis Cardio Center, Niguarda Hospital Milan Italy; ^5^ Division of Cardiac Surgery Fondazione IRCCS Policlinico San Matteo Pavia Italy; ^6^ Department of Medicine and Surgery University of Milan‐Bicocca Milan Italy; ^7^ Cardiothoracic and Vascular Department (DICATOV) IRCCS Ospedale Policlinico San Martino Genova Italy

**Keywords:** functional mitral regurgitation, heart failure, MitraClip, pulmonary hypertension, transcatheter mitral valve repair

## Abstract

**Background:**

Pulmonary hypertension (PH) is frequently observed in patients with functional mitral regurgitation (FMR) and heart failure with reduced ejection fraction (HFrEF) and adversely impacts prognosis. However, limited data exist on the outcomes of transcatheter edge‐to‐edge mitral valve repair (M‐TEER) in patients with PH, particularly regarding hemodynamic subtypes.

**Methods:**

This multicenter, retrospective analysis included 144 HFrEF patients with moderate‐to‐severe or severe FMR who underwent M‐TEER across four Italian centers. Baseline hemodynamic assessment was performed using right heart catheterization (RHC) in conscious patients. Procedural outcomes and clinical follow‐up were evaluated at 1 year. The endpoints studied included death from any cause, heart failure hospitalization and a composite endpoint of both.

**Results:**

Among the 144 patients, 84% had PH (64% combined post‐ and pre‐capillary‐PH (Cpc‐PH), 20% isolated post‐capillary‐PH (Ipc‐PH)). Procedural success was achieved in 92%, with significant improvements in New York Heart Association (NYHA) functional class (*p* < .001) and echocardiographic parameters. At 1 year, the composite endpoint occurred in 30% of patients, with higher rates in PH patients compared to no PH group (34% vs. 9%, respectively, *p* = .039). Among PH patients, Cpc‐PH patients demonstrated the worst outcomes (for the composite endpoint at 1 year Cpc‐PH 37% vs. Ipc‐PH 24% vs. no‐PH 9%, *p* = .031). Multivariate analysis confirmed Cpc‐PH as a significant predictor of adverse outcomes at 1 year.

**Conclusions:**

M‐TEER is an effective therapeutic option for patients with HFrEF and FMR, providing significant procedural success and clinical improvements. However, patients with PH, particularly those with Cpc‐PH, exhibit worse long‐term clinical outcomes.

## Graphical abstract

This study reveals that patients with PH have worse clinical outcomes, in terms of hospitalization for heart failure (HF) and the composite endpoint of HF hospitalization and death at 1‐year follow‐up, compared with patients without PH. In particular, the hemodynamic subgroup of combined post‐ and pre‐capillary PH is associated with worse clinical outcomes. Hemodynamic assessment with right heart catheterization at baseline in patients with FMR who are candidates for M‐TEER may yield important information and guide treatment decisions.

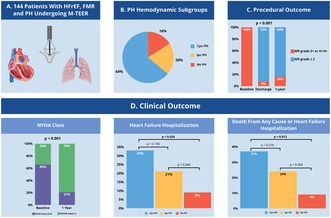

## 1 INTRODUCTION

Transcatheter edge‐to‐edge mitral valve repair (M‐TEER) has emerged as a key therapeutic option for patients with symptomatic functional mitral regurgitation (FMR).^1^ This minimally invasive procedure has shown significant benefits in improving clinical outcomes, including reducing heart failure (HF) hospitalizations, improving survival and enhancing quality of life.^2^, ^3^, ^4^ A recent meta‐analysis of the three larger randomized clinical trials conducted in this field—СОАPT (Cardiovascular Outcomes Assessment of the MitraClip Percutaneous Therapy for Heart Failure Patients with Functional Mitral Regurgitation),^2^ MITRA‐FR (Percutaneous Repair with the MitraClip Device for Severe Functional/Secondary Mitral Regurgitation)^5^ and RESHAPE‐HF2 (A Randomized Study of the MitraClip Device in Heart Failure Patients With Clinically Significant Functional Mitral Regurgitation)^3^ trial—has further confirmed the positive impact of MitraClip on clinical outcomes in this population.^1^, ^6^

While existing trials provided valuable evidence, patients with concomitant pulmonary hypertension (PH) have been systematically under‐represented. However, PH is common in HF patients with FMR, as a long‐term increase in left‐sided filling pressure due to mitral regurgitation (MR) often leads to PH, which increases the risk of worsening HF, congestive symptoms and mortality.^7^, ^8^ While PH has been associated with adverse outcomes post‐M‐TEER, the role of distinct hemodynamic phenotypes assessed by right heart catheterization (RHC) remains poorly defined. In particular, differentiating between isolated post‐capillary PH (Ipc‐PH) and combined post‐ and pre‐capillary PH (Cpc‐PH) could provide valuable insights into disease severity and prognosis, yet invasive data in this context are scarce.

This study investigates the 1‐year clinical outcomes of M‐TEER in patients with HFrEF, FMR and PH, focusing on the impact of different hemodynamic subtypes of PH.

## 2 METHODS

### 2.1 Patient selection and definitions

This is a multicentric, retrospective analysis of a prospective data collection including all consecutive HFrEF patients affected by moderatetosevere or severe (3+ or 4+/4+) FMR, who consecutively underwent M‐TEER between January 2013 and December 2022 in four centers in Italy (Vito Fazzi Hospital in Lecce, IRCCSS San Matteo Hospital in Pavia, Niguarda Hospital in Milan, IRCCS San Martino Hospital in Genova).

At baseline, an analysis of RHC was conducted in conscious patients to assess hemodynamic status. All RHCs were performed within 4 weeks prior to the M‐TEER procedure, and no changes in medical therapy were made in the interim. The following hemodynamic parameters were collected: pulmonary artery wedge pressure (PAWP); systolic, diastolic and mean PAP (sPAP, dPAP and mPAP); CO evaluated with thermodilution; cardiac index (CI); right atrial pressure (RAP); pulmonary vascular resistance (PVR). PAWP was measured at end‐expiration in the absence of breath hold, gated to the EKG QRS complex. Transthoracic and transesophageal echocardiography were performed according to guidelines to evaluate the technical feasibility of the procedure.^9^, ^10^ A transthoracic echocardiogram was repeated at a 1‐year follow‐up. All patients included in the study were receiving maximized guideline‐directed optimal medical therapy at the time of the Heart Team evaluation. Enrolled patients were not involved in the study design; however, they played a central role in providing baseline information and regarding the use of procedural and outcome data for research purposes.

In line with the 2022 ESC Guidelines on Pulmonary Hypertension,^11^ PH was defined as a mean pulmonary arterial pressure (mPAP) >20 mmHg. Furthermore, patients were classified based on the type of PH: Cpc‐PH was defined by an mPAP >20 mmHg, a pulmonary artery wedge pressure (PAWP) >15 mmHg, and a pulmonary vascular resistance (PVR) >2 Wood units (WU); Ipc‐PH was defined as an mPAP >20 mmHg, a PAWP >15 mmHg, and a PVR ≤2 WU.

FMR was defined as MR resulting from incomplete leaflet coaptation due to left ventricular remodelling.

M‐TEER was performed with the MitraClip system (Abbott Vascular), under general anaesthesia using transesophageal echocardiography and fluoroscopic guidance, as previously described.^12^

Procedural success was defined as the achievement of residual MR ≤ 2, in the absence of procedure failure, which was characterized by the abortion of the procedure or conversion to open surgery at the end of the procedure.

### 2.2 Data collection and follow‐up

Baseline clinical, echocardiographic, laboratory and invasive hemodynamic characteristics were collected, along with procedural and post‐procedural data. Clinical follow‐up data were primarily obtained during scheduled outpatient visits. When follow‐up data were not available, information was gathered through telephone interviews with patients or their family members, or from registration offices, primary care physicians and rehospitalization records. Outcomes were assessed at 1‐year follow‐up from the date of the procedure and included echocardiographic and clinical data, such as death from any cause, HF hospitalization, heart transplantation and a composite endpoint of death from any cause or HF hospitalization rate. HF hospitalization was defined as rehospitalization in the presence of onset or worsening of symptoms or signs secondary to abnormal cardiac function.

The study protocol adheres to the ethical principles outlined in the 1975 Declaration of Helsinki. Written informed consent was obtained from all patients at all participating centers.

### 2.3 Statistical analysis

Results are shown as mean ± standard deviation (SD) for normally distributed continuous variables (normality distribution assessed by visual inspection of the quantile‐quantile plot), as median with 25th and 75th percentiles for nonnormally distributed continuous variables, and as percentages for categorical data. One‐way analysis of variance (ANOVA) and Student's unpaired *t*‐test were applied to compare normally distributed continuous variables. For nonnormally distributed continuous variables, the Kruskal–Wallis test and Wilcoxon rank‐sum test were used to compare data between three and two groups, respectively. Chi‐square and Fisher's exact tests were used for categorical variables comparison. Survival was reported using the Kaplan–Meier method, with comparisons made using the log‐rank test. A two‐tailed *p* value of <.05 was considered statistically significant. Univariate analysis of predictors of the outcomes analysed was conducted using Cox proportional hazards regression. Left ventricular ejection fraction (LVEF), age and procedural success (which had *p* values <.10 at univariate analysis) were included in a multivariate Cox regression model to evaluate the hazard ratio (HR) and 95% confidence interval (CI) of the relationship between predictors and outcomes. All statistical analyses were performed using R software (version 3.4.4, http://www.R‐project.org).

## 3 RESULTS

### 3.1 Study population and procedural outcomes

A total of 144 patients were enrolled, with a mean age of 67.8 ± 10.2 years. The majority of patients were male (71%); 94 patients (66%) presented with NYHA class III or IV. The mean LVEF was 29.4% ± 9.3%, and the mean RHC‐derived mPAP was 31.6 ± 10.8 mmHg. A total of 122 patients (84%) with evidence of PH: 64% met the definition of Cpc‐PH and 20% of Ipc‐PH. Baseline characteristics, including clinical, echocardiographic and hemodynamic data, are summarized in Table S1.

All patients underwent implantation of at least one clip (1.9 ± .5 clips per patient). Procedural success was achieved in 92% of patients (Table S2). Pre‐discharge B‐natriuretic peptide was reduced compared to baseline (*p* = .032) (Table S2).

### 3.2 Overall clinical outcomes

All patients completed 1 year of follow‐up. After 12 months, 121 (84%) patients had MR ≤2. No significant changes were noted in HF drug dosages compared to baseline (Table S3). The proportion of patients classified as NYHA class III/IV decreased from 66% at baseline to 14% (*p* < .001). Tricuspid annular plane systolic excursion/systolic pulmonary artery pressure (TAPSE/sPAP) ratio significantly improved from .39 ± .16 to .46 ± .18 (*p* = .015) (Table S3).

**FIGURE 1:**
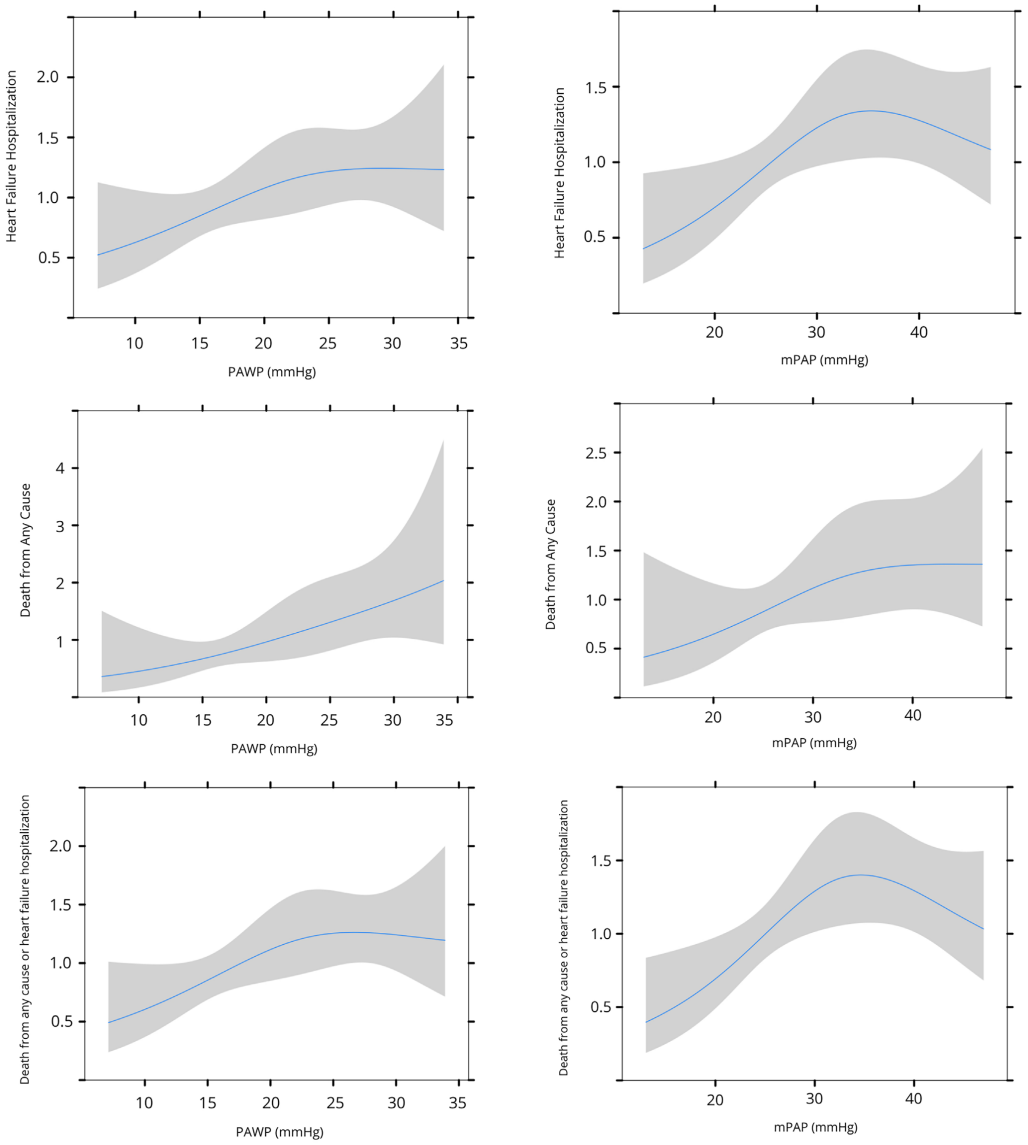
Unadjusted association between baseline mPAP and PAWP as continuous variables and the relative hazard of 1‐year death from any cause, HF rehospitalization and composite end point of both in all patients. Shaded areas represent the 95% CIs for the HR at each pressure. HF, heart failure; mPAP, mean pulmonary artery pressure; PAWP, pulmonary artery wedge pressure.

At 1‐year, death from any cause occurred in 9% of patients, with 27% of patients experiencing rehospitalization for HF (Table S4). The composite endpoint (death from any cause or hospitalization for HF) was observed in 30% of patients (Table S4). Figure 1 and Figure S1 show the relationship between baseline mPAP and PAWP with clinical outcomes at follow‐up.

### 3.3 Pulmonary Hypertension Subgroup Analysis

Compared with patients with a normal hemodynamic profile, patients with PH presented at baseline with a higher rate of ischemic cardiomyopathy (*p* = .028), reduced LVEF (*p* = .006), reduced TAPSE/sPAP ratio (<.001) and higher values of the main surgical risk scores (Table 1).

**TABLE 1:** Baseline and procedural differences between PH and no‐PH subgroups.

	Pulmonary hypertension (*n* 122)	No pulmonary hypertension (*n* 22)	*p* Value
Age, years	69 (52–75)	66.5 (62–76.2)	.737
Male, %	92 (75)	10 (45)	.006
Diabetes, %	27 (22)	4 (18)	.785
Hypertension, %	58 (47)	8 (36)	.462
COPD, %	16 (13)	3 (14)	1
CKD, %	63 (52)	11 (50)	1
EGFR, mL/min/m^2^	61.4 (±22.8)	62.6 (±20.1)	.663
Previous AMI, %	56 (46)	5 (23)	.069
Ischemic cardiomyopathy, %	61 (50)	5 (23)	.028
Furosemide, mg/die	52.5 (37.5–100)	50 (25–75)	.437
Hb, g/dl	12.6 (11.3–13.7)	12.6 (11.2–14.2)	.833
Euroscore log	9.2 (6.2–13.2)	4.6 (2.5–7.3)	.036
Euroscore II	5.2 (2.5–7.4)	3.2 (2.1–5.1)	.036
STS mortality	4.8 (2.7–4)	3.8 (1.9–5.2)	.138
STS morbidity	20.3 (14.9–30.8)	13 (9.9–20.8)	.024
LVEDV, ml	245.1 (±84.7)	205.6 (±73.4)	.047
LVEF, %	27 (23–32.2)	31 (28–38)	.006
TAPSE/PASP	.3 (.3–.4)	.5 (.5–.7)	<.001
Procedural success, %	110 (90)	20 (91)	.313
1‐year success, %	102 (84)	20 (91)	.573

Abbreviations: AMI, acute myocardial infarction; CKD, chronic kidney disease; COPD, chronic obstructive pulmonary disease; eGFR, estimated glomerular filtration rate; Hb, haemoglobin; LVEDV, left ventricular end‐diastolic volume; LVEF, left ventricular ejection fraction; PH, pulmonary hypertension; STS, Society of Thoracic Surgeons; TAPSE/PASP, TAPSE to systolic pulmonary artery pressure ratio.

During follow‐up, patients with PH experienced significantly worse clinical outcomes compared to those without PH (Table 2). After 1 year, the composite endpoint of death from any cause or hospitalization for HF was higher in PH patients (34% vs. 9%, *p* = .039). Adjusted multivariable Cox regression showed PH as a significant predictor of the composite endpoint of death or hospitalization (adjusted HR 8.10, 95% CI: 1.11–60.50, *p* = .039) and of HF hospitalization (adjusted HR 7.78, 95% CI: 1.05–57.50, *p* = .044) (Table S5).

**TABLE 2:** Clinical outcomes between PH and no‐PH subgroups at 1 year.

	Pulmonary hypertension (*n* 122)	No pulmonary hypertension *n* (22)	*p* Value
Death, %	13 (11)	1 (4)	.695
HF hospitalization, %	37 (30)	2 (9)	.071
Composite endpoint (death + HF hospitalization), %	41 (34)	2 (9)	.039

Abbreviation: HF, heart failure.

### 3.4 Pulmonary hypertension: Hemodynamic subgroups

**FIGURE 2:**
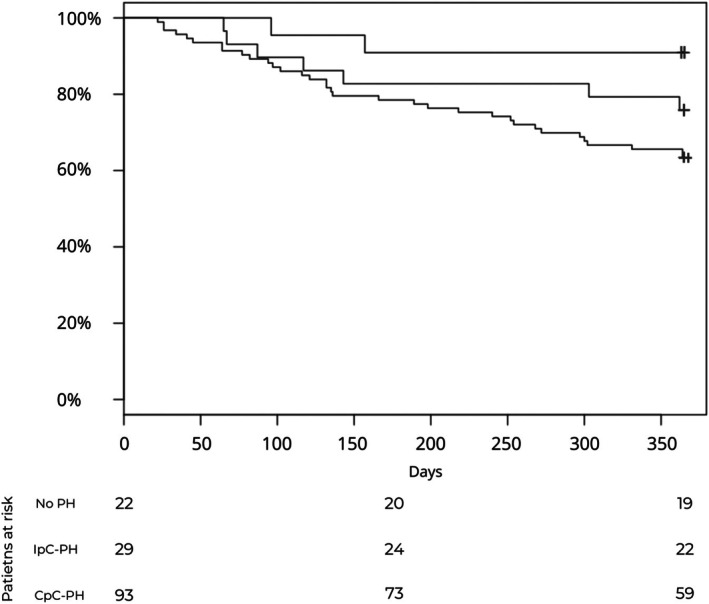
Kaplan–Meier curves for freedom from composite end point (death from any cause or HF rehospitalization) according to PH subgroup. The number of patients at risk is reported at baseline, 182 days and 365 days for each subgroup. HF, heart failure; PH, pulmonary hypertension. ||, no pulmonary hypertension; +, isolated post‐capillary pulmonary hypertension; ++, combined post‐ and pre‐capillary pulmonary hypertension.

Baseline characteristics of hemodynamic subgroups are summarized in Table S6. At 1‐year follow‐up, the composite endpoint of death or HF hospitalization occurred in 37% of patients with Cpc‐PH, 24% of those with Ipc‐PH and only 9% of patients without PH (*p* = .012). This difference was primarily driven by HF hospitalizations, which were significantly higher in the Cpc‐PH group (33%) compared to no‐PH (9%, *p* = .024), while no significant difference was observed between Ipc‐PH and no‐PH (21% vs. 9%, *p* = .440) (Table 3). Figure 2 shows Kaplan–Meier curves for freedom from composite endpoint for the different hemodynamic subgroups.

**TABLE 3:** Clinical outcomes at 1 year according to PH hemodynamic subgroups.

	Cpc‐PH (*n* 93)	Ipc‐PH (*n* 29)	No PH (*n* 22)	*p* Value		
				Cpc‐PH vs. No‐PH	Ipc‐PH vs. No‐PH	Cpc‐PH vs. Ipc‐PH
Death, %	10 (11)	3 (10)	1 (4)	.688	.625	.709
HF hospitalization, %	31 (33)	6 (21)	2 (9)	.024	.440	.196
Composite endpoint (death + HF hospitalization), %	34 (37)	7 (24)	2 (9)	.012	.268	.216

Abbreviations: Cpc‐PH, combined post‐capillary and pre‐capillary pulmonary hypertension; HF, heart failure; Ipc‐PH, isolated post‐capillary pulmonary hypertension; PH, pulmonary hypertension.

Multivariable analyses confirmed that Cpc‐PH was an independent predictor of adverse outcomes at 1‐year follow‐up (adjusted HR 9.4, 95% CI: 1.3–69.5, *p* = .028 for the composite endpoint; adjusted HR 8.7, 95% CI: 1.2–65.0, *p* = .034 for HF hospitalization). No significant differences were found between Cpc‐PH and Ipc‐PH in terms of HF hospitalization (adjusted HR: 1.72, 95% CI: .65–4.54, *p* = .270) and the composite endpoint (adjusted HR: 1.92, 95% CI: .73–5.26, *p* = .185) (Table 4).

**TABLE 4:** Unadjusted and adjusted hazard ratios for the probability of HF hospitalization and the composite endpoint of HF hospitalization and mortality according to pulmonary hypertension hemodynamic subgroups at 1‐year follow‐up.

1 year follow‐up		Univariate analysis		Multivariate analysis	
		HR (95% CI)	*p* Value	HR (95% CI)	*p* Value
Hospitalization for heart failure	Cpc‐PH vs. No‐PH	4.30 (1.03–18.1)	.044	8.70 (1.20–65.00)	.034
	Ipc‐PH vs. No‐PH	2.40 (.50–11.90)	.285	5.1 (.60–43.70)	.140
	Cpc‐PH vs. Ipc‐PH	1.81 (.76–4.35)	.185	1.72 (.65–4.54)	.270
Composite EP (Death or Hospitalization)	Cpc‐PH vs. No‐PH	4.70 (1.10–19.60)	.033	9.40 (1.30–69.50)	.028
	Ipc‐PH vs. No‐PH	2.80 (.60–13.80)	.187	4.90 (.60–42.20)	.148
	Cpc‐PH vs. Ipc‐PH	1.60 (.72–3.70)	.234	1.92 (.73–5.26)	.185

Abbreviations: Cpc‐PH, combined post‐capillary and pre‐capillary pulmonary hypertension; EP, endpoint; Ipc‐PH, isolated post‐capillary pulmonary hypertension; PH, pulmonary hypertension.

## 4 DISCUSSION

The results of the present study show that in patients with significant FMR undergoing M‐TEER, the procedure is effective, resulting in sustained improvements in functional status and a good procedural success rate at 1‐year follow‐up. However, patients with PH, particularly those with Cpc‐PH, exhibited worse outcomes, with higher rates of hospitalization for HF and a higher incidence of the composite endpoint of death from any cause or hospitalization for HF.

Data regarding the relationship between preoperative PH and outcomes after M‐TEER are limited, particularly in patients affected by FMR. Randomized clinical trials focused on M‐TEER did not specifically investigate the prognostic relevance of PH. A post hoc analysis of the COAPT trial found that elevated echocardiographically derived sPAP was associated with higher mortality and heart failure hospitalization at 2 years, regardless of treatment; however, MitraClip combined with optimal medical therapy reduced the incidence of the composite endpoint (death and HF hospitalization) compared to medical therapy alone, with benefit seen across all sPAP levels.^13^ It is important to underline that PH was diagnosed based on transthoracic echocardiographic measurements, which do not provide a complete characterization of the patient's hemodynamics and pathophysiology. Transthoracic echocardiography may have further limitations: in cases of significant tricuspid regurgitation, it may lead to an underestimation of the right ventricular systolic pressure; additionally, even when right ventricular systolic pressure is accurately measured, it may not fully represent the true extent of pulmonary pressure, particularly in the presence of severe right ventricular dysfunction or low cardiac output. As such, relying solely on Doppler‐derived right ventricular systolic pressure for diagnosing PH may result in misdiagnosis or an inaccurate assessment of its severity.^14^, ^15^

A retrospective cohort study analysed more than 4000 patients (across 232 US sites in the STS/ACC TVT registry) who underwent M‐TEER with the MitraClip system (Abbott Vascular), stratifying them into four groups according to baseline invasive mPAP.^16^ At 1‐year follow‐up, PH was associated with higher mortality and increased rates of HF re‐hospitalization, with the risk of adverse events rising progressively as mPAP increased, even after multivariable adjustment (HR per 5 mmHg mPAP increase, 1.05; *p* = .02). Similarly, a single‐institution study showed that PH, defined by RHC, was associated with higher mortality after M‐TEER.^17^ The study also found that the 2022 PH definition, with an mPAP >20 mmHg, better predicted survival outcomes than the 2015 guidelines definition (mPAP >25 mmHg), as even mild increases in mPAP were linked to a significantly worse prognosis. However, these studies involved both patients with degenerative and FMR, where the pathophysiologic and hemodynamic contributions of MR—and thus the hemodynamic effect of mitral valve intervention—may differ. Additionally, the hemodynamic evaluations were assessed during the procedure, under general anaesthesia and eventually under varying doses of inotropic and vasopressor medications, which may significantly influence the hemodynamic profile.^18^

Our study, focusing only on FMR patients and assessing hemodynamic profile by RHC in conscious patients, found that PH increases the risk of adverse clinical outcomes during follow‐up after M‐TEER. In particular, among PH patients, those with Cpc‐PH exhibited the highest rates of hospitalization for HF and of the composite endpoint of death or HF hospitalization. This could be explained by the fact that Cpc‐PH may indicate a more advanced degree of disease, with pulmonary vascular remodelling and heightened right ventricular afterload which, when persistent, can adversely affect right ventricular function. These findings are in line with recent studies that have shown a correlation between elevated PVR and invasive parameters of right ventricular dysfunction with worse outcomes following M‐TEER.^17^, ^19^ Overall, these findings suggest that Cpc‐PH in this setting could be a marker of advanced cardiopulmonary disease, which may contribute to poorer outcomes despite procedural success.

We believe that our data do not show the futility of M‐TEER in patients with PH. Although PH patients are burdened with worse clinical outcomes, previous data have shown that both Ipc‐PH and Cpc‐PH patients can still experience significant hemodynamic improvements after M‐TEER.^20^ In particular, patients with Ipc‐PH can even be able to achieve normalization of pulmonary and wedge pressures.^20^ In addition, it has also been suggested that such hemodynamic improvements may at least in part be predicted by the response to vasodilator testing during RHC at baseline.^21^ For these reasons, considering that PH is associated with a worse prognosis, but that it cannot be considered a single pathology, but a continuum with different degrees of severity, performing baseline hemodynamic assessment with RHC in FMR patients who are candidates for M‐TEER may provide valuable insights into pathophysiology and prognosis, and help guide treatment decisions. In particular, identification of Cpc‐PH can help recognize patients at higher risk of adverse outcomes in whom earlier intervention—before the development of advanced right ventricular dysfunction or irreversible pulmonary vascular remodelling—might be considered. Conversely, patients with Ipc‐PH may still derive substantial benefit from M‐TEER, supporting timely referral once indications are met. Regarding the possible clinical benefits of M‐TEER compared to optimal medical therapy in the setting of FMR with PH, more clinical data are certainly needed.

This study has some limitations that need to be acknowledged. Indeed, this is an observational study based on a retrospective analysis of prospectively collected data, inherently limited by lack of randomization in exposure. Furthermore, this is a multicenter study reporting clinical practices across different centers; therefore, clinical and procedural data have been reported by the various sites and investigators without core lab adjudication. Moreover, the small number of patients without PH may have reduced statistical power for comparisons with PH subgroups: our results need to be confirmed by studies with larger samples. Finally, the study population consisted of patients from a single ethnic and geographic background, which may limit the generalizability of our findings.

## 5 CONCLUSION

M‐TEER represents an important therapeutic option for patients with HFrEF and FMR, providing significant procedural success and clinical improvements. However, patients with PH, particularly those with Cpc‐PH, exhibit worse long‐term clinical outcomes.

## AUTHOR CONTRIBUTIONS

Alessandro Mandurino‐Mirizzi designed and performed the research, analysed the data and drafted the manuscript. Luca Raone, Andrea Raffaele Munafò, Fabrizio Gazzoli, Marco Mussardo, Claudio Montalto and Francesco Germinal collected data from each centre and revised the manuscript. Marco Ferlini, Italo Porto, Giuseppe Colonna, Jacopo Oreglia, Gabriele Crimi supervised the process and were responsible for the final manuscript revision.

## CONFLICT OF INTEREST STATEMENT

All authors have no conflict of interest to declare.

## Supporting information


Appendix S1.

## Data Availability

The data that support the findings of this study are available from the corresponding author upon reasonable request.
