# Supplementary material for: Clinical outcomes of transcatheter edge‐to‐edge repair in patients with functional mitral regurgitation and pulmonary hypertension
Source: Eur J Clin Invest. 2025 Oct 3;56(1):e70130. doi: 10.1111/eci.70130 (PMC12817235; doi:10.1111/eci.70130)

**Supplementary Table 1**

| **Baseline characteristics and procedural data (n=144)** | |
| --- | --- |
| Age | 67.8 (±10.2) |
| Male, % | 102 (71) |
| Hypertension, % | 66 (46) |
| Diabetes, % | 31 (21) |
| Smoke, % | 61 (42) |
| Dyslipidemia, % | 81 (56) |
| PAOD, % | 12 (8) |
| COPD, % | 19 (13) |
| CKD , % | 74 (51) |
| Previous AMI, % | 61 (42) |
| Previous PCI, % | 65 (45) |
| Previous CABG, % | 26 (18) |
| Previous stroke, % | 8 (5) |
| Post-ischemic HF, % | 66 (46) |
| ICD, % | 90 (62) |
| CRT, % | 28 (19) |
| NYHA class III-IV, % | 94 (66) |
| Atrial fibrillation, % | 51 (35) |
| Admission for HF in the last year, % | 96 (67) |
| 3C-HF score, % | 22.0 (±17.9) |
| Euroscore Log, % | 11.1 (±9.8) |
| Euroscore II, % | 5.6 (±4.1) |
| STS mortality, % | 5.1 (±3.7) |
| STS morbidity, % | 22.4 (±11.7) |
| LVEDD, mm | 66.4 (± 12.3) |
| LVEDV, ml | 238.9 (± 84.0) |
| LVEF, % | 29.4 (± 9.3) |
| EROA, cm2 | 0.35 (± 0.15) |
| TAPSE, mm | 17.1 (± 3.3) |
| TAPSE/sPAP ratio | 0.39 (± 0.16) |
| Implanted clip/pts | 1.9 (±0.5) |
| Procedural success (MR ≤2), % | 131 (92) |
| ACE/ARB, % | 135 (94) |
| Beta-blocker, % | 128 (89) |
| Furosemide, % | 135(94) |
| Furosemide, mg/die | 82.2 (±38.4) |
| MRA, % | 106 (74) |
| Nitrates, % | 25 (17) |
| P2Y12 inhibitors, % | 73 (51) |
| Oral anticoagulant, % | 78 (54) |
| Inotropes, % | 21 (14) |
| IABP, % | 7 (5) |
| PH, % | 115 (80) |
| Cpc-PH, % | 82 (57) |
| IpC-PH, % | 33 (23) |
| CI, l/min/m2 | 1.82 (±0.42) |
| PAWP, mmHg | 21.4 (±8.0) |
| mPAP, mmHg | 31.6 (±10.8) |
| RAP, mmHg | 7.2 (±4.3) |
| PVR, UW | 3.5 (±1.9) |

*PAOD: Peripheral Artery Occlusive Disease. COPD: Chronic Obstructive Pulmonary Disease. CKD: Chronic Kidney Disease. AMI: Acute Myocardial Infarction. PCI: Percutaneous Coronary Intervention. CABG: Coronary Artery Bypass Graftin.g HF: Heart Failure. ICD: Implantable Cardioverter Defibrillator. CRT: Cardiac Resynchronization Therapy. NYHA: New York Heart Association. 3C-HF: Cardiac Comorbidities, Chronic Heart Failure Score. STS: Society of Thoracic Surgeons. LVEDD: Left Ventricular End-Diastolic Diameter. LVEDV: Left Ventricular End-Diastolic Volume. LVEF: Left Ventricular Ejection Fraction. EROA: Effective Regurgitant Orifice Area. TAPSE: Tricuspid Annular Plane Systolic Excursion. sPAP: Systolic Pulmonary Artery Pressure. ACE: Angiotensin-Converting Enzyme. ARB: Angiotensin II Receptor Blockers. MRA: Mineralocorticoid Receptor Antagonists. P2Y12: Platelet ADP P2Y12 Receptor Inhibitors. IABP: Intra-Aortic Balloon Pump. PH: Pulmonary Hypertension. Cpc-PH: Combined Post-Capillary and Pre-Capillary Pulmonary Hypertension. Ipc-PH: Isolated Post-Capillary Pulmonary Hypertension. CI: Cardiac Index. PAWP: Pulmonary Artery Wedge Pressure. mPAP: Mean Pulmonary Artery Pressure. RAP: Right Atrial Pressure. PVR: Pulmonary Vascular Resistance.*

**Supplementary Table 2.** Baseline vs pre-discharge data

|  | **Before procedure** | **After procedure** | **least square mean of differences** | **P value** |
| --- | --- | --- | --- | --- |
| **N patients=144** | | | | |
| BNP, pg/ml | 2459 (4864-54) | 1768 (2274-1262) | -264 (+53.9;-293.9) | 0.032 |
| eGFR, ml/min/m2 | 60.6 (91-29) | 63.0 (97-29) | + 2.4 (-1.3; +6.1) | 0.203 |
| Hb, g/dl | 13.2 (±1.4) | 11.6 (±1.7) | - 1.33 (-1.05; - 1.62) | <0.001 |
| MR grade 3+ or 4+/4+, % | 144 (100) | 13 (8) | -92% | <0.001 |
| Mean transmitral gradient, mmHg | 1.87 (±0.67) | 3.35 (±1.26) | + 0.96 (+0.10;+1.81) | 0.029 |

*BNP: B-Type Natriuretic Peptide. eGFR: Estimated Glomerular Filtration Rate. Hb: Hemoglobin. MR: Mitral Regurgitation.*

**Supplementary Table 3**. Baseline vs 1-year follow-up data

|  | **Before procedure** | **1 year follow-up** | **least square mean of differences** | **p- value** |
| --- | --- | --- | --- | --- |
| **N patients=144** | | | | |
| NYHA class III/IV, % | 94 (66) | 21 (14) | -52% | <0.001 |
| Furosemide, mg/die | 82.2 (±38.4) | 81.4 (±81.6) | - 1.3 (+ 2.6; -20.7) | 0.757 |
| LVEDV, ml/m2 | 238.9 (± 84.0) | 226.7 (±79.8) | - 5.8 (- 15.5; +3.9) | 0.187 |
| LVEF, % | 29.4 (± 9.3) | 28.0 (±9.9) | - 1.6 (-0.1; -3.1) | <0.001 |
| MR grade 3+ or 4+/4+, % | 144 (100) | 23 (16) | -84% | <0.001 |
| Mean trans-mitral gradient, mmHg | 1.87 (±0.67) | 3.35 (±1.26) | + 0.96 (+0.10;+1.81) | 0.029 |
| TAPSE, mm | 17.1 (± 3.3) | 17.8 (± 3.4) | + 1.0 (+0.1; +2.0) | 0.035 |
| PASP, mmHg | 49.5 (14.9) | 42.1 (13.4) | - 7.2 (-3.6; -10.7) | <0.001 |
| TAPSE/sPAP ratio | 0.39 (± 0.16) | 0.46 (±0.18) | + 0.06 (+0.01;+0.11) | 0.015 |
| TR grade 3+ or 4+/4+, % | 11 (8) | 7 (5) | -92% | 0.784 |

*NYHA: New York Heart Association. LVEDV: Left Ventricular End-Diastolic Volume. LVEF: Left Ventricular Ejection Fraction. MR: Mitral Regurgitation. TAPSE: Tricuspid Annular Plane Systolic Excursion. PASP: Pulmonary Artery Systolic Pressure. TAPSE/sPAP: TAPSE to Systolic Pulmonary Artery Pressure Ratio, TR: tricuspid Regurgitation.*

**Supplementary Table 4.** Clinical outcomes

| **1 year- follow-up** | |
| --- | --- |
| Death, % | 14 (9) |
| HF hospitalization, % | 39 (27) |
| Composite endpoint (death + HF hospitalization), % | 43 (30) |
| Heart transplantation, % | 5 (3) |

*HF: Heart failure*

**Supplementary Table 5.** Multivariable Cox proportional hazards models adjusted for Age, ischemic heart disease and procedural success (which had p values <0.10 at univariate analysis) for the association between pulmonary hypertension and clinical endpoints: HF hospitalization and the composite endpoint of HF hospitalization and mortality.

| **1 year follow-up** | **Univariate Analysis** | | **Multivariate Analysis** | |
| --- | --- | --- | --- | --- |
|  | **HR (95% CI)** | **P value** | **HR (95% CI)** | **P value** |
| **Hospitalization for heart failure** | 1.34 (0.92-15.80) | 0.065 | 7.78 (1.05-57.50) | 0.044 |
| **Composite EP (Death or Hospitalization)** | 4.20 (1.02-17.50) | 0.035 | 8.10 (1.11-60.50) | 0.039 |

*EP: Composite Endpoint, HR: Hazard Ratio.*

**Supplementary Table 6.** Baseline and procedural differences between hemodynamic subgroups.

|  | **Cpc-PH**  **(n 93)** | **Ipc-PH**  **(n 29)** | **No PH**  **(n 22)** | **P value** |
| --- | --- | --- | --- | --- |
| Age, years | 68.5 (±10.5) | 66.2 (±10.0) | 67.5 (±9.5) | 0.567 |
| Male, % | 68 (75) | 24 (83) | 10 (45) | **0.008** |
| Diabetes, % | 21 (23) | 6 (21) | 4 (18) | 0.896 |
| Hypertension, % | 44 (47) | 14 (48) | 8 (36) | 0.623 |
| COPD, % | 13 (14) | 3 (10) | 3 (14) | 0.937 |
| CKD, % | 48 (52) | 15 (52) | 11 (50) | 0.99 |
| EGFR, ml/min/m2 | 60.9 (±23.8) | 58.5 (±19.1) | 62.6 (±20.1) | 0.808 |
| Previous AMI, % | 45 (49) | 11 (38) | 5 (23) | 0.07 |
| Ischemic cardiomyopathy, % | 47 (50) | 14 (48) | 5 (23) | 0.116 |
| Furosemide, mg/die | 40 (25-100) | 62.5 (40-100) | 50 (25-75) | 0.636 |
| Hb, g/dl | 12.6 (11.3-13.7) | 12.8 (11.6-13.9) | 12.6 (11.2-14.2) | 0.836 |
| Euroscore log | 8.4 (6.1-14.4) | 9.9 (6.5-11) | 4.6 (2.5-7.3) | 0.103 |
| Euroscore II | 5.3 (2.5-7.4) | 5.0 (2.6-7.4) | 3.2 (2.1-5.1) | 0.111 |
| STS mortality | 4.8 (2.7-8.0) | 4.0 ( 1.5-7.1) | 3.8 (1.9-5.2) | 0.275 |
| STS morbidity | 22.3 (14.9-33.1) | 18.0 (15.6-23.0) | 13 (9.9-20.8) | 0.053 |
| LVEDV, ml | 241 (±86.1) | 257 (81) | 205.6 (±73.4) | 0.094 |
| LVEF, % | 27 (23-33) | 27 (24-31) | 31 (28-38) | **0.023** |
| TAPSE/PASP | 0.3 (0.3-0.4) | 0.4 (0.3-0.5) | 0.5 (0.5-0.7) | **<0.001** |
| Procedural success, % | 82 (88) | 28 (96) | 20 (91) | 0.157 |
| 1-year success, % | 76 (82) | 26 (90) | 20 (91) | 0.23 |

*Cpc-PH: Combined Post-Capillary and Pre-Capillary Pulmonary Hypertension. Ipc-PH: Isolated Post-Capillary Pulmonary Hypertension. PH: Pulmonary Hypertension. COPD: Chronic Obstructive Pulmonary Disease. CKD: Chronic Kidney Disease. eGFR: Estimated Glomerular Filtration Rate. AMI: Acute Myocardial Infarction. Hb: Hemoglobin. STS: Society of Thoracic Surgeons. LVEDV: Left Ventricular End-Diastolic Volume. LVEF: Left Ventricular Ejection Fraction. TAPSE/PASP: TAPSE to Systolic Pulmonary Artery Pressure Ratio.*

**Supplementary Figure 1.** Receiver operating characteristic (ROC) curves for the prediction of clinical outcomes based on pulmonary artery wedge pressure (PAWP) and mean pulmonary artery pressure (mPAP) for the entire study population.


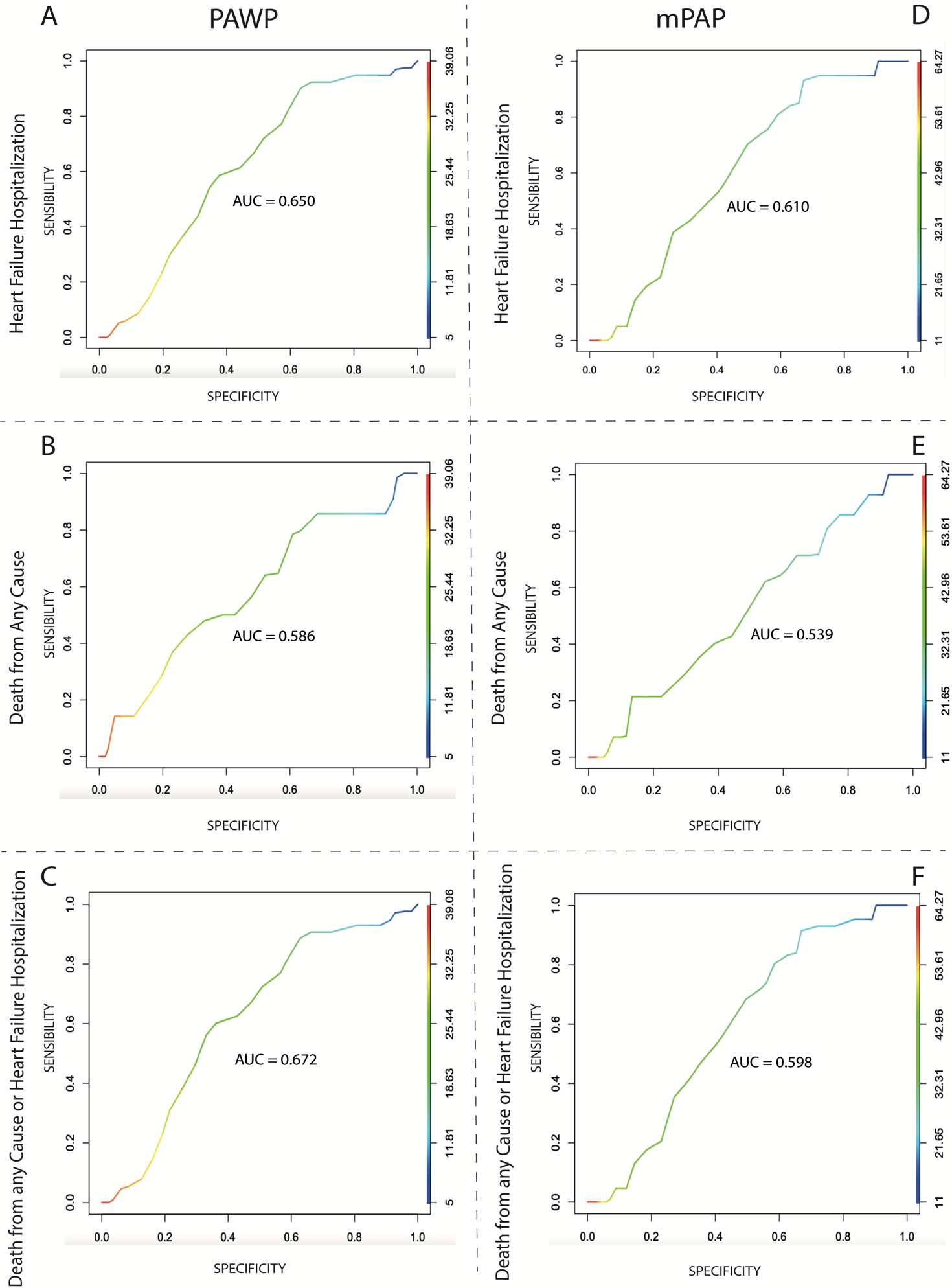

Supplement: Supplementary file 1 — Appendix S1. [file ECI-56-e70130-s001.docx]
